# An Analysis of the Progression of Conjunctivalisation after Transplantation of Cultivated Corneal Epithelium

**DOI:** 10.1155/2021/8499640

**Published:** 2021-11-22

**Authors:** Dariusz Dobrowolski, Boguslawa Orzechowska-Wylegala, Bogumil Wowra, Ewa Wróblewska-Czajka, Maria Grolik, Edward Wylegala

**Affiliations:** ^1^Chair and Clinical Department of Ophthalmology, Division of Medical Science in Zabrze, Medical University of Silesia in Katowice, Panewnicka 65 St., 40760 Katowice, Poland; ^2^Department of Ophthalmology, St. Barbara Hospital, Trauma Centre, Medykow Square 1, 41200 Sosnowiec, Poland; ^3^ENT Department, John Paul II Upper Silesian Child Health Centre, Division of Medical Science in Katowice, Medical University of Silesia, Medyków 16 Str., 40752 Katowice, Poland

## Abstract

**Purpose:**

To analyse the recurrence of superficial neovascularisation after previous corneal surface reconstruction with cultivated corneal epithelial cells.

**Materials and Methods:**

Forty-eight eyes underwent autologous transplantation of cultivated corneal epithelium to treat partial or total limbal stem cell deficiency caused by chemical or thermal injury. The carrier for the epithelial sheets was a denuded amniotic membrane. Follow-up was conducted for up to 120 months. Recurrent revascularisation (measured in terms of clock hours affected) was evaluated with slit-lamp examination and the support of confocal microscopy.

**Results:**

During the long-term observation, only 7 eyes had stable epithelia with no neovascularisation from the conjunctiva. Nineteen eyes developed pathologic vessels in 1 quadrant, with additional 4 eyes developing them in 2 quadrants. Twelve patients developed subtotal or total conjunctivalisation of the corneal surface. They were referred for second cultivated epithelium transplantation (3 patients), allogenic keratolimbal transplantation (7 patients), or keratoprosthesis (2 patients). Six patients withdrew consent. The use of confocal scans of up to 100 *µ*m in resolution enabled the detection of pathologic microvasculature originating from the conjunctiva and the exclusion of stromal vascular ingrowth.

**Conclusions:**

Local ingrowth of the conjunctiva is a common complication after the transplantation of cultivated epithelial cells. Severe and progressive vascularisation inevitably leads to graft failure. However, if local ingrowth stops before reaching the central cornea, the treatment even with this complication can be considered a success.

## 1. Introduction

Limbal stem cell deficiency (LSCD) is defined as a lack of corneal epithelium renewal caused by damage or severe injury to the corneal epithelial stem cells of the limbus [1]. In this condition, unhealed sites of epithelial erosion corresponding to limbal injury are covered by the surrounding conjunctival tissue. This causes corneal haze, vascularisation, and permanent ocular inflammation and discomfort. It is a complex disorder involving various degrees of severity; it may be local, partially covering the peripheral cornea, or it may cover the whole surface of the cornea with conjunctiva.

Depending on the cause, the disease can be divided into primary and secondary manifestations. The most common causes of the primary form of the disease are aniridia, ectodermal dysplasia, sclerocornea, hormonal disorders, and keratitis-ichthyosis-deafness (KID) syndrome. These diseases are congenital and are associated with mutations or disorders in the organogenesis of ocular tissues.

Secondary causes of corneal LSCD are diagnosed much more often than primary causes are. The most common cause is the chemical or thermal burn of the ocular surface. Other causes are Stevens-Johnson syndrome and ocular pemphigoid. LSCD may occur in the pterygium, as a complication of neurotrophic keratopathy, in contact lens wearers or in patients who have undergone surgery affecting the corneal limbus. Inflammatory processes involving the limbal zone can damage stem cells that regenerate the corneal epithelium and replace the conjunctiva.

The standard method of treating limbal failure involves removing abnormal conjunctival tissue and replacing it with regenerating corneal epithelial cells. For a few years, the treatment of choice for this condition has been the transplantation of cultured corneal epithelium [2]. This is perceived as the optimal method because it is minimally invasive for the donor eye and does not require excess surgery on the surface of the involved eye. A small piece of the limbus, which can be obtained from the eye that is partially damaged, is sufficient to restore the stratified corneal epithelium. The epithelial material must meet several criteria to allow for efficient renewal of epithelial cells on the damaged surface.

Autologous transplantation of cultivated corneal epithelium is a procedure with confirmed effectiveness; moreover, it results in satisfactory improvement of visual acuity and reduction of ocular discomfort [3, 4]. This approach minimises surgical intervention and makes the entire procedure repeatable. One of the most common complications of surgery is revascularisation with local regrowth of conjunctival pannus. LSCD recurrence can be caused by decreased epithelial renewal ability, as well as inflammation, which is one of the strongest factors stimulating conjunctival neovascularisation [5]. In addition, conjunctival pannus can recur years after surgery, suggesting a slow loss of proliferative potential of the transplanted epithelium over a period of several years [6].

The aim of this study was to determine in detail the progression of conjunctival vascularisation appearing after many years of follow-up. To assess the success rate of the procedure, we compared the number of clock hours of conjunctival neovascularisation with the extent of limbal involvement before surgery and conducted follow-up on patients for up to 120 months. Such data are important for determining grafts' lifespans. The correlation of conjunctival neovascularisation with the degree of limbal damage should help to assess the risk of LSCD recurrence in the postoperative period.

## 2. Materials and Methods

The study was performed in accordance with the tenets of the Declaration of Helsinki. Signed written informed consent was obtained from all patients before any procedures were carried out.

The team of researchers prepared for 5 years by cultivating corneal epithelia from cadaveric donors. After obtaining repeatable results, we received funding from the Ministry of Science and approval from the bioethical commission for clinical application in humans. The team responsible for the laboratory part and cell culture included Dr Dobrowolski, Dr Orzechowska-Wylegala, Dr Wowra, and Dr Grolik; the team that performed transplantation of the cultured epithelium consisted of Dr Dobrowolski and Professor Wylegala. Finally, the postoperative follow-up was evaluated by Dr Dobrowolski, Dr Wowra and Dr Wroblewska-Czajka. All surgeries were performed in the Clinical Department of Ophthalmology of the Medical University of Silesia in Katowice, Poland.

### 2.1. Cultivation Technique

The cultivated corneal epithelium was prepared by our team in the laboratory of the Experimental Pharmacology Unit of the Medical University of Silesia in Katowice. Briefly, the tissue source was the healthy eye of each patient. With a crescent knife, a 2 mm 2 biopsy of limbal tissue from the top of the Vogt palisades was gently cut. Biopsies were taken from the superior limbus. The tissue was treated with dispase for 30 min and then trypsinised with 1% Trypsin with 0.01% EDTA for 10 min to obtain a cell suspension. Cells were gently scraped with a microscraper. A cellular suspension with a density of 1–4 × 104 cells per 1 ml was settled in the culture medium. Cultures were carried out in the presence of a feeder layer of 3T3 fibroblasts located underneath the cell carrier. Denuded amniotic membrane was the proper carrier of the cultured corneal epithelium.

Cultures were carried out in standard conditions at 37°C in a humidified atmosphere of 5% CO2 and 95% air. The medium was supplemented by a DMEM/HAM F12 mixture with 10% bovine serum, 0.5% dimethyl sulfoxide (DMSO), 10 ng/ml of mouse epidermal growth factor (EGF), 5 *μ*g/ml of bovine insulin, 0.1 nM cholera toxin, 0.18 mM adenine, 2 nM triiodothyronine, 4 mM L-glutamine, 0.4 mg/ml of hydrocortisone, and 100 *μ*g/ml of penicillin and streptomycin mixture. The culture medium was changed every 48 h. On the 10th day of culturing, plates were evaluated under a light microscope to evaluate epithelial growth.

Histological evaluation focused on epithelial layer regularity and the number of cell layers. Haematoxylin-eosin staining was performed for analysis. The corneal epithelium was then immunostained for cytokines 3, 12, and 19; protein p63; and connexin 43 [7, 8]. Obtaining a stratified, multilayer epithelium, positive assessment of its morphology and histochemical confirmation of its origin allowed the tissue to be used clinically. The presence of the p63 protein demonstrated proliferative potential.

### 2.2. Study Group Characteristics

The study included 48 patients (46 men and 2 women) whose ages ranged from 13 to 69 years at the time of surgery. Forty-four patients were injured by alkaline agents, 2 were injured by acidic agents, and 2 experienced thermal burns caused by steam. Each patient in the study presented limbal stem cell deficiency with total or partial conjunctival pannus; severity was defined according to Dua's six-degree LSCD scale and involvement of the limbus in clock hours [9] (Table 1). All patients were injured at least 2 years prior to the limbal transplant. In all cases, one eye was injured, and the patient's unaffected eye was used as a source of limbal epithelial cells for cultivation and further reconstruction of the corneal epithelium.

To define the study group, the following criteria were applied. Inclusion criteria were limbal insufficiency accompanied by conjunctival ingrowth over the corneal stroma with superficial vasculature of conjunctival origin. In terms of inclusion criteria, patients were previously disqualified from autologous limbal transplants if they had a range of LSCD over 6 or more clock hours of the limbus involved. Another inclusion criterion was the central involvement of the visual axis and surrounding area. This region was defined as a central area of 6 mm in diameter that was covered by pathological tissue from the conjunctiva.

Signs of active inflammation or progressing neovascularisation on the surface excluded patients from the study, as did severe damage to the corneal stroma (stromal thinning below 400 *μ*m; deep vasculature in the stroma). Other exclusion criteria included cicatrisation or keratinisation of the conjunctival fornix that required plastic surgery of the conjunctiva and palpebra, uncontrolled glaucoma poorly responding to topical treatment, and severe dryness of the eye.

We conducted surgery with one of the three following grades depending on the severity of the limbal deficiency: grade IV with 6–9 clock hours of limbal involvement, grade V with 9–12 hours of limbal injury, and grade VI with total limbal deficiency. In cases of conjunctival involvement, cases with less than 6 clock hours were referred to autologous transplants or superficial keratectomy.

### 2.3. Surgical Procedure

The surgeries were performed from 2009 to 2011. All patients underwent transplantation of cultivated autologous corneal epithelium, which was applied to cover the area of the previously removed conjunctival pannus. The carrier for the corneal epithelium was denuded amniotic membrane [10]. Patients were operated on under local anaesthesia. First, a 180–360° peritomy was conducted to establish the new conjunctival margins; then, the conjunctiva was fixed 1–2 mm behind the limbal area with 10–0 nylon sutures. Subsequently, the conjunctival pannus was gently removed with a spatula or crescent knife. Amniotic carriers with epithelial cells were fitted on the denuded corneas. Peripheral continuous or single 10–0 nylon sutures were applied to stabilise the graft just beyond the limbal border to the adjacent sclera. A bandage contact lens was placed on the corneal surface at the end of the surgery for no more than 7 days. The amniotic scaffolds were not removed after the procedure, and they were integrated with the exposed corneal tissue. A new border was formed between the corneal epithelium and the conjunctival epithelium, and this border was assessed in the following months of observation [11].

Postoperative treatment consisted of 14 days of topical fluoroquinolone treatment administered five times a day and dexamethasone therapy with the following scheme: seven times a day for 14 days, five times a day for 14 days, three times a day for 30 days, and once a day for 30 days. Oral prednisone was taken at a dose of 20 mg for 14 days and then 10 mg for the next 14 days.

During the first 6 months after surgery, postoperative follow-ups were more frequent. Patients were seen at 1 week, 4 weeks, 8 weeks, and 13 weeks after surgery. Following this, visits took place every 6 months.

### 2.4. Outcome Measures

Follow-up was conducted for up to 120 months. The main outcome measure was the presence of recurrent conjunctival pannus as measured by the number of clock hours of fibrovascular involvement of the limbus and the surrounding corneal surface. The success criterion was the lack of recurrence of the conjunctival tissue within the visual axis, even if it crossed the border of the corneal limbus and partially covered the cornea, sparing the visual axis. If the tissue reached the corneal axis again, we described the graft as a failure. The invasion of the conjunctival epithelium into the central part of the cornea was not an obstacle for further evaluation. If the invasion was local, partial LSCD with central involvement was not a contraindication against penetrating keratoplasty. Continued observation was focused on conjunctival invasion over the transplanted tissue.

The secondary assessment was the follow-up of the treated patients and the eligibility for other therapeutic methods as a continuation of treatment or alternative treatment to the failure of the original treatment method. Further limbal grafts were planned in patients who developed symptoms of complete failure. Another option was switching to keratoprosthesis in the remaining patients when the conjunctival invasion persisted despite our best therapeutic efforts.

## 3. Results

**Figure 1:**
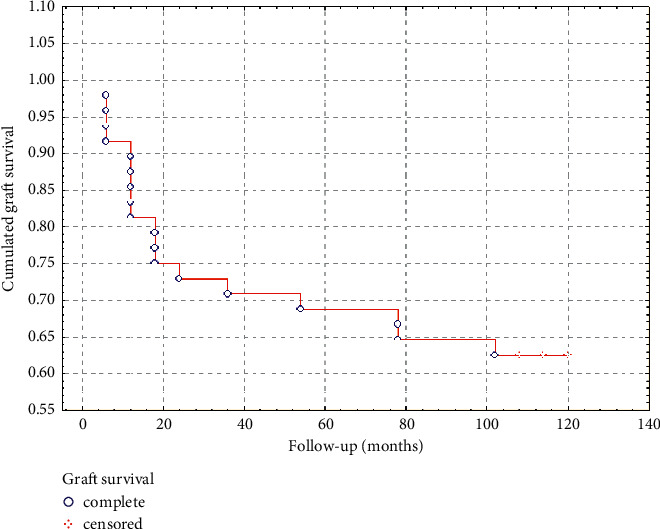
Kaplan–Mayer curve showing graft survival rate.

The nature of the corneal damage caused by burns makes it extremely difficult to achieve stabilisation of the ocular surface. Concordantly, of our 48 patients, only 7 experienced complete resolution of conjunctival vascularisation. In fact, only a few patients achieved the expected effect of the corneal centre and its periphery not being occupied by conjunctival tissue. In most of the remaining patients, we observed local growth of the conjunctiva without the involvement of the centre of the cornea. The Kaplan–Mayer curve shows the progression of vascularisation in our patients (Figure 1). In the group of treatment failures, pannus regrowth occurred early and was already observed shortly after the transplant had been performed. Extensive anti-inflammatory treatment did not improve outcomes in these failures but only reduced the regrowth rate of the conjunctival tissue over the surface of the cornea. As such, a possible consequence of conjunctival pannus restoration is the need to repeat the procedure or resort to a different treatment, such as an allogeneic transplant or keratoprosthesis implantation.

The main endpoint measured was the progression of vascular invasion from the corneal periphery. The area between the transplanted epithelium and conjunctiva is known to be active during the observation period. In follow-up control visits, many eyes presented with reconstruction with conjunctival invasion, as well as the formation of stable epithelial margins. In the long term, only seven eyes exhibited stable epithelia with the absence of vascular ingrowth from the conjunctiva. Among these eyes, the conjunctival margin at the periphery was regular, and no epithelial defects or erosions were noted. Nineteen eyes developed pathologic vessels in one quadrant; however, conjunctival ingrowth was localised, with typical involvement of 1-2 clock hours. Four eyes developed neovascularisation in two quadrants. Thirty eyes showed no pathological tissue within the visual axis, although the conjunctiva crossed the limbal border in 23 cases. Thus, the success criterion was met. The other eyes with conjunctival growth from at least three quadrants had the visual axes occupied as follows. Nine eyes suffered from conjunctival invasion in three quadrants, and nine developed subtotal or total conjunctivalisation of the surface. These data indicate that the conjunctiva crossing the limbal boundaries in three or more quadrants (or at least 6 clock hours of the limbus) causes invasion involving the visual axis.  Table 2 presents the detailed range of conjunctival ingrowth in each 6-month period of follow-up.

Patients with local conjunctival invasion accompanied by stromal haze were candidates for keratoplasty. These procedures were performed in six patients with invasion in one corneal quadrant and two patients with invasion of the conjunctival epithelium in two quadrants. The operations were dictated by scarring changes under the epithelium located in the central part of the cornea, including those involving the visual axis.

Because of the long duration of treatment, six patients declined and withdrew consent for further follow-up and observation. Patients who discontinued participation in the study did not object to the use of existing medical data; they only refused to continue participating in the study group, undergo diagnostic procedures, or participate in further observation and ambulatory visits. Follow-up of these patients was terminated when further participation was refused. With their consent, the obtained data are presented in the paper.

**Figure 2:**
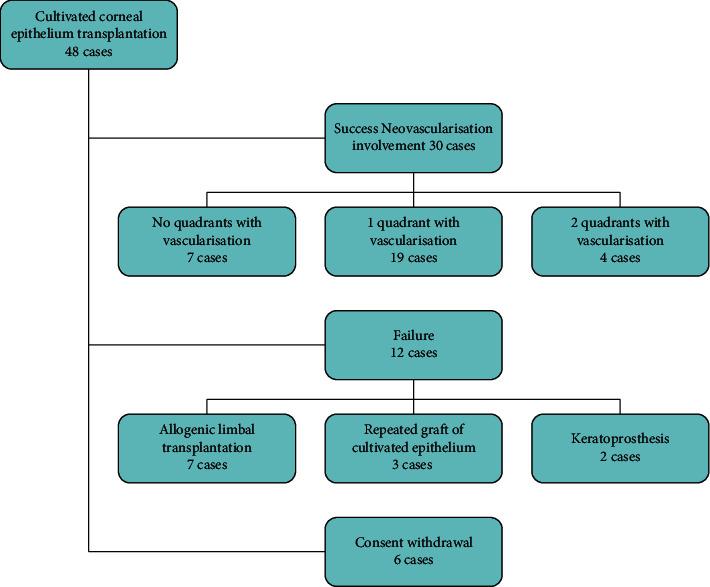
Diagram of patients' follow-up.

Among the patients who underwent subsequent phases of the intervention, 12 qualified for further treatment, which was performed because of failure of the primary procedure. These treatments included allogenic grafts (seven eyes), reoperation with cultivated cells (three eyes), and keratoprosthesis implantation (two eyes).  Figure 2 delivers a general description of patients' follow-up.

**Figure 3:**
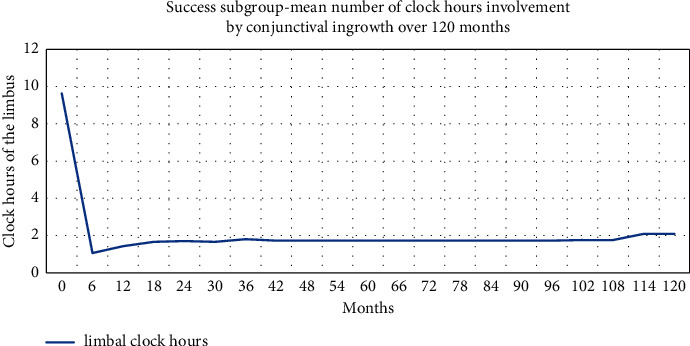
Average of clock hours of conjunctival invasion in success group over 120 months.

Most patients presented with slight corneal haze caused by the amnion, but this haze subsided in the following months. However, in three eyes, amniotic tissue margins were clearly visible throughout the follow-up period. In 62.5% of eyes, there was no recurrent conjunctival neovascularisation reaching the area of the pupil. Otherwise, in most patients, the limbal area was irregular, with some vessels in the limbal region. It is accepted that cultured epithelium transplantation cannot restore regular limbal margins. However, such irregularities were not considered to be a conjunctival invasion of the corneal surface. In patients who achieved therapy success, the visual axis was not occupied.  Figure 3 shows how the average number of clock hours of limbus occupation involved the conjunctival tissue without reaching the central part of the cornea.

**Figure 4:**
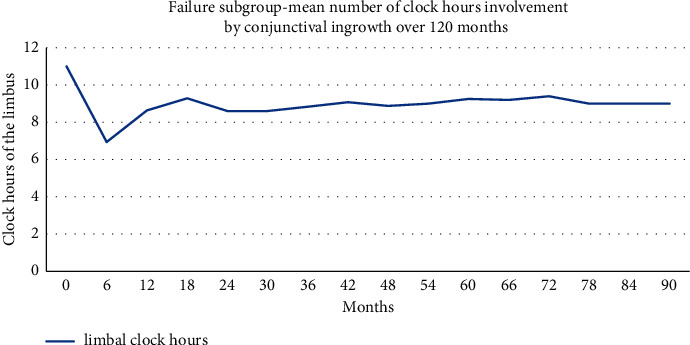
Average of clock hours of conjunctival invasion in failure group over 120 months.

We considered eyes to be graft failures in cases of superficial revascularisation of the corneal centre (visual axis involvement) and/or stromal scarring with new stromal vessels. We observed that 25% of eyes met the graft failure criteria; they remained cloudy because of central revascularisation or stromal haze (Figure 4). However, only six eyes (12.5%) developed total conjunctival pannus covering the entire cornea.

**Figure 5:**
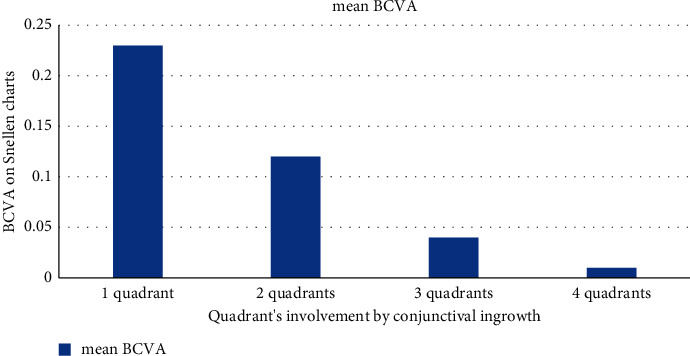
Mean visual acuity obtained by patients depending on the degree of conjunctival recurrence in the consecutive corneal quadrants.

Visual acuity strongly corresponded to the smoothness of the central epithelium; vascularisation close to the pupil resulted in epithelial irregularity and a reduction in vision quality such that visual acuity ranged from counting fingers to 0.6. However, a stable corneal surface did not guarantee good quality of vision. The visual acuity results of corneas with vascularisation exceeding no more than one quadrant ranged from 0.1 to 0.6. Disturbances caused by amniotic membrane haze, irregular astigmatism, or irregular stromal shape reduced the quality of vision. In two cases, improvement was achieved by the application of scleral lenses (maximum best corrected visual acuity of 0.6). With an increasing number of quadrants involved, visual acuity was limited by the proximity of the pannus, affecting the visual axis. Two quadrants of conjunctival ingrowth reduced visual acuity to a maximum of 0.4; if a third quadrant was involved, visual acuity decreased to 0.1 or less (Figure 5). All these cases eventually became failures. We note that 25% of cases, excluding total LSCD, failed because of the involvement of the visual axis.

**Figure 6:**
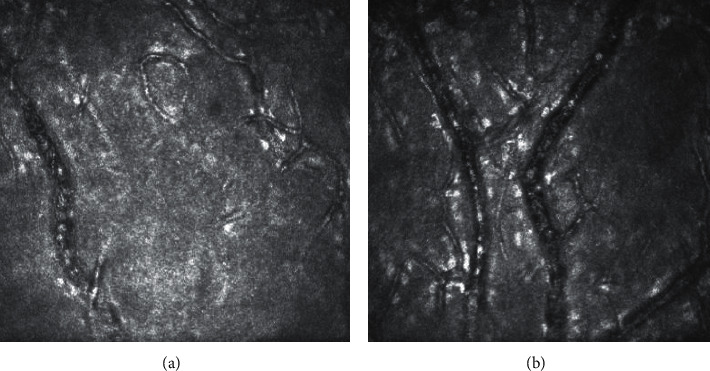
Analysis of corneal vascularisation in moderate (a) and advanced (b) conjunctival ingrowth assessed with confocal microscopy support.

In confocal microscopy analysis involving 6 mm of the central cornea, we observed that five eyes had stable epithelia and had no vessels in the epithelia or anterior stroma. Another two eyes were affected by a single vascular ingrowth from the conjunctiva. Of the 19 eyes with pathological vasculature in one quadrant, conjunctival ingrowth reaching the paracentral area was present in five eyes. Finally, four eyes that developed neovascularisation in two quadrants exhibited single vessels reaching the central area. The number of clock hours of peripheral ingrowth did not correlate with the severity of central cornea involvement. Thus, the appearance of vessels even in the central part of the cornea was not synonymous with their visualization under a slit lamp. Confocal microscopy permits the identification of such vessels when scanning the central and paracentral parts of the cornea (Figure 6). It seems that they do not affect the surface of the corneal epithelium and cannot be treated as an objective symptom of limbal failure. Likewise, they do not disqualify the patient from achieving the success criteria. The cause of their appearance may be inflammatory or related to extensive surgical intervention. During the postoperative period, most of these vessels tend to disappear without threatening patients' vision. Long-term observation of conjunctival ingrowth on the cornea indicates that the surface remains stable and does not have the features of recurrence that may have otherwise affected the patients' outcomes.

## 4. Discussion

Studies on the effectiveness of treating limbal failure suggest that the delivery of such tissue may support residual cells remaining in the limbus. It has been confirmed that, during allogeneic transplantation, donor genetic material is not found despite the stabilisation of the corneal surface and the disappearance of disease features [12]. This may indicate that the cultured cells support or stimulate the remaining limbal cells of the host. This is one of the factors influencing the procedure's effectiveness in cases of partial or even total limbal deficiency.

The effectiveness of restoring the corneal epithelium with autologous cells can be confirmed by cytologic examination or confocal microscopy [13, 14]. The percentage of conjunctival and corneal epithelial cells allows the determination of the degree of deficiency of corneal epithelial cells, which allows us to indirectly check the effectiveness of our technique. Another solution is to assess one of the typical features of patients with limbal insufficiency. Long-term evaluation of conjunctival invasion after limbal cell transplantation is noninvasive; with such an approach, we can monitor the degree of LSCD in the long term and determine whether the procedure was successful. This also eliminates the need to perform numerous cytological tests or obtain limbal biopsies. The confocal microscopy used in diagnostics also provides some help, which allows us to verify the degree of conjunctival epithelium ingrowth over the cornea and differentiate it from the deep vascular ingrowth in the corneal stroma. In our protocol, we used epithelial tissues grown on an amniotic membrane substrate. This choice arose from the need to use a carrier that could be easily transferred to the surface of the treated eye. Thanks to the presence of a basement membrane, amniotic sheets can be easily sewn to the surface of the damaged eye. Another variant of this method that is currently recommended is the use of a fibrin gel scaffold as a matrix to maintain the cell culture.

Pellegrini et al. described the method of cultivation and transplantation of epithelial cells on fibrin membranes [15]. Other papers have described several additional methods, such as restoring the epithelial layer using amniotic membranes as the epithelial carrier, delivering a cell suspension, and cultivating oral mucosa epithelial cells. A classical approach involves conjunctival-limbal autografts and keratolimbal allografts (CLAU and KLAL) and family-related keratolimbal allografts (lr-KLAL) as effective treatments [16, 17]. The concept of this approach is the same, which is to deliver healthy cells and to let them restore the anatomy of the corneal surface. The application of epithelial cells results in the prevention of persistent epithelial defects, achievement of epithelial regularity, and reduction of conjunctival vascularisation and inflammatory infiltration. For patients, this results in improved ocular comfort and long-term visual acuity. Such methods as KLAL are radical; therefore, minimisation of surgery is expected. As a result, cell cultivating methods are constantly evolving [18].

The LSCD grade depends on factors that interfere with the ocular surface epithelium. Most commonly, ocular chemical burns lead to multifocal and complicated lesions. The severity of thermal burns depends on the temperature and duration of exposure. Such damage affects not only the corneal epithelium but also all layers of the ocular surface, resulting in treatment complexity, which usually necessitates long-term management of the patient's ocular surface disorders. Final success depends on proper treatment in the acute phase, precise qualification for ocular reconstruction with adequate surgical methods, and further complex postoperative approaches [19].

The results of the cultured epithelium transplantation are highly dependent on the preoperative condition of the recipient's eye [20]. The conjunctival pannus should be superficial or limited to minimal stromal involvement. A good indication for surgery is an absence of or minimal stromal vascularisation. Deep vessels spread throughout the stroma suggest severe burn history, cicatrisation, stromal thinning, and the risk of an inadequate inflammatory response after transplantation. In eyes with stromal vessels present, we observed further extension of vessels within the stroma despite anti-inflammatory treatment. This happened even though stromal keratectomy was performed to remove the vascularised tissue. Limbal ischaemia and uncontrolled revascularisation were observed in eyes with ocular surface involvement of grade V or VI. The presence of inferior and superior fornices of the conjunctiva is important for proper lubrication of the surface and adequate topical drug delivery. The involvement of the conjunctiva with synechiae reduces the success rate. In general, stabilisation of the corneal surface after a burn injury must be confirmed before the procedure because inflammation or cicatrisation can lead to graft failure. Hence, it is clear that all elements of the ocular surface must be evaluated for qualification [21].

Another prognostic factor is the area of limbal involvement. According to Paulkin et al., corneal surface restoration can be achieved in 83.3% of eyes with partial limbal deficiency and in 63.3% of eyes with total limbal involvement [22]. Our results show that the number of failures rose as the grade of ocular burn increased. Stromal and conjunctival revascularisation was characteristic of burns of grade V or VI and was much less common in burns of lower grades. Rama et al. observed eyes for 10 years after cultivated epithelium transplantation and found a success rate (i.e., the rate of maintenance of epithelial renewal) of 76.6%. However, they also showed that graft failure was quite common in such cases. The final success was influenced by the type and severity of the ocular damage and postoperative complications [23]. Nakamura et al. described different applications of cultivated epithelia. Such applications can be effective for chemical injuries, as well as for severe cases of Stevens-Johnson syndrome or ocular pemphigoid [24]. The success rate of epithelial transplants varies significantly depending on the underlying disease and its severity. Shimazaki et al. reported that in severe ocular surface injuries, such as chemical burns and Stevens-Johnson syndrome, the success rate was 65%. Tissues that are autologous, allogeneic, or derived from the patient's relatives are good sources for effective treatment. An alternative technique is the use of oral mucosa epithelial cultures, which are useful in bilateral pathologies [25].

Gander et al. compared the results of the autologous epithelial cell culture procedure between adults and children [26]. Their results are promising because total success was achieved in 80.9% of adults and 66.6% of children. However, it should be noted that improvements in the quality of vision were only seen in a smaller proportion of patients; an increase in visual acuity of at least two lines was only observed in 21.2% of children and in 38% of adult cases.

The use of confocal microscopy to assess corneal vascularisation is more effective than using other imaging methods. In particular, slit-lamp photographs do not allow for the measurement of the median number of vessels. When standardised, confocal scans allow the measurement of the number of intersecting vessels. In addition, they can be used to visualize the surrounding cells, including inflammatory or immunocompetent cells, which can in turn confirm the existence of coinflammation [27]. However, confocal microscopy is rarely used in cases of corneal neovascularisation. Most studies have focused on the assessment of lymphatic vessels in the cornea, which are deeper than the superficial blood vessels of the corneal epithelium or stroma [28, 29].

The analysis performed highlights the value of the surgery because it allowed for the quantitative assessment of tissue [30]. This complements qualitative assessments that assess visual acuity. The measurement of the number of vessels may have prognostic significance and can indicate the presence of an inflammatory process that requires anti-inflammatory treatment. The presence of proinflammatory factors does not depend only on the presence of epithelium but probably also on the extent of tissue damage. Vascular monitoring may be helpful in establishing treatments to prevent recurrence of primary vascularisation.

Our results show that careful qualification and examination before surgery can protect patients from graft failure. A qualifying examination excludes patients with an active inflammatory process, progressive neovascularisation, or advanced injuries of the conjunctiva and eyelids; therefore, this procedure is crucial to identify patients with the predominant presence of superficial vascularised conjunctival tissue over the cornea. This main criterion causes the cultured epithelium to replace the conjunctival tissue, which is the basic assumption of the treatment. This approach limits the group of patients admitted to the study but gives the group homogeneity. The data presented increases the value of the Dua ocular involvement scale in the evaluation of patients before limbal transplantation. It is also important to examine the transplant after the procedure. Conjunctival epithelial invasiveness and in vivo degree of vascularisation in confocal microscopy are important outcomes used to assess the healing process. They allow early detection of pathological vessels to ensure adequate treatment [31].

The main conclusion of our study is that the stability of the epithelium on the corneal surface depends on a low degree of conjunctival invasion. Conversely, vascularisation of three or four quadrants always results in ingrowth of the conjunctiva over the central cornea, causing the patient to be assessed as a treatment failure. Undoubtedly, the smallest possible growth of the conjunctiva was a guarantee of epithelial stability. This conclusion applies to patients with a lack of conjunctival vascularisation, as well as patients with only one quadrant involved. These patients had the highest rates of success, and this is the basis for the conclusion that stabilisation in cases of mild invasion of the conjunctival epithelium affecting a maximum of 4 clock hours may be considered a criterion for the long-term success of cultured grafts. In contrast, in cases where the central zone is progressively occupied by the conjunctival epithelium and results in the loss of optical function, the first thing to consider is a repeat of the cell culture transplant. The worst outcome in this case is a huge extensity of conjunctival growth when there is a complete failure of the corneal limbus and relapse to the original condition. In these cases, allogeneic transplants from a related person or an unrelated donor can be considered, and the last resort is to offer these patients keratoprosthesis implantation as a treatment.

Overall, it is worth emphasising that the cell culture method should be the method of choice for mild or moderate burns. Although severe ocular burns can still be treated with cultivated epithelia, alternative methods, such as keratoprosthesis implantation, should be considered first. In addition, the risk of failure seems to increase with burn severity and primary limbal destruction.

## Figures and Tables

**Table 1:** Types of ocular burns and involvement of corneal surface in Dua's scale and in clock hours of the limbus involvement in each patient.

Patient	Burn	Burning agent	Dua scale rate	Initial involvement of the limbus in clock hours
1	Thermal	Steam	IV	7
2	Chemical	Alkaline	V	11
3	Chemical	Alkaline	IV	8
4	Chemical	Alkaline	V	11
5	Chemical	Alkaline	V	10
6	Chemical	Alkaline	V	11
7	Chemical	Acidic	IV	8
8	Chemical	Alkaline	VI	12
9	Chemical	Alkaline	V	11
10	Chemical	Alkaline	VI	12
11	Chemical	Alkaline	VI	12
12	Chemical	Alkaline	V	3
13	Chemical	Alkaline	V	9
14	Chemical	Alkaline	V	11
15	Chemical	Alkaline	VI	12
16	Chemical	Alkaline	V	10
17	Chemical	Alkaline	VI	12
18	Chemical	Alkaline	VI	12
19	Chemical	Alkaline	V	11
20	Chemical	Alkaline	V	11
21	Chemical	Alkaline	V	11
22	Chemical	Alkaline	IV	8
23	Chemical	Alkaline	IV	8
24	Chemical	Alkaline	V	10
25	Chemical	Alkaline	V	11
26	Chemical	Alkaline	V	11
27	Chemical	Alkaline	V	11
28	Chemical	Acidic	IV	8
29	Chemical	Alkaline	IV	7
30	Chemical	Alkaline	V	10
31	Chemical	Alkaline	VI	12
32	Chemical	Alkaline	VI	12
33	Chemical	Alkaline	VI	12
34	Chemical	Alkaline	V	9
35	Chemical	Alkaline	V	10
36	Chemical	Alkaline	V	11
37	Chemical	Alkaline	VI	12
38	Chemical	Alkaline	V	10
39	Thermal	Steam	V	9
40	Chemical	Alkaline	V	11
41	Chemical	Alkaline	V	9
42	Chemical	Alkaline	VI	12
43	Chemical	Alkaline	IV	7
44	Chemical	Alkaline	IV	6
45	Chemical	Alkaline	VI	12
46	Chemical	Alkaline	VI	12
47	Chemical	Alkaline	V	11
48	Chemical	Alkaline	V	11

**Table 2:** Patients' overview range of conjunctival ingrowth (clock hours) in each 6-month period of follow-up.

Pat.	Dua scale rating		0	6	12	18	24	30	36	42	48	54	60	66	72	78	84	90	96	102	108	114	120
			Months after surgery, R-rejected from the study (retransplantation or consent withdrawal) number of clock hours of conjunctival neovascularisation on the corneal surface (0-initial number of clock hours of the cornea covered by conjunctiva)																				
1	IV		7	0	0	0	0	0	0	0	0	0	0	0	0	0	0	0	0	0	0	0	0
2	V		11	0	1	2	2	2	2	2	2	2	2	2	2	2	2	2	2	2	2	2	2
3	IV		8	0	2	3	3	3	3	3	3	3	3	3	3	3	3	3	3	3	3	3	3
4	V		11	2	5	6	6	6	6	7	7	7	8	8	9	R							
5	V		10	0	0	1	1	1	1	1	1	1	1	1	1	1	1	1	1	1	1	1	1
6	V		11	3	4	4	4	4	4	4	4	4	4	4	4	4	4	4	4	4	4	4	4
7	IV		8	1	1	1	1	2	3	3	3	3	3	3	3	3	3	3	3	3	3	3	3
8	VI		12	10	12	12	12	12	12	12	12	12	12	R									
9	V		11	3	3	4	4	4	4	4	4	4	4	4	4	4	4	4	4	4	4	4	4
10	VI		12	8	9	10	10	10	12	12	12	12	12	12	12	R							
11	VI		12	12	12	12	12	R															
12	V		3	7	7	7	7	7	7	7	7	7	7	7	7	R							
13	V		9	0	1	2	2	2	2	2	2	2	2	2	2	2	2	2	2	3	3	3	3
14	V		11	0	0	0	0	0	0	0	0	0	0	0	0	0	0	0	0	0	0	0	0
15	VI		12	4	4	4	5	1	1	1	1	1	1	1	1	1	1	1	1	1	1	1	1
16	V		10	0	1	1	1	1	1	1	1	1	1	1	1	1	1	1	1	1	1	1	1
17	VI		12	6	9	9	9	9	9	9	9	9	R										
18	VI		12	8	8	8	8	8	8	8	8	8	8	R									
19	V		11	2	2	2	2	2	2	2	2	2	2	2	2	2	2	2	2	2	2	2	2
20	V		11	4	4	4	4	4	4	4	4	4	4	4	4	4	4	4	4	4	4	4	4
21	V		11	12	12	12	12	R															
22	IV		8	3	3	3	3	3	3	3	3	3	3	3	3	3	3	3	3	3	3	3	3
23	IV		8	2	2	2	2	2	4	4	4	4	4	4	4	4	4	4	4	4	4	4	4
24	V		10	1	1	1	1	1	1	1	1	1	1	1	1	1	1	1	1	1	1	1	1
25	V		11	7	9	9	9	9	9	9	R												
26	V		11	0	1	2	2	2	2	2	2	2	2	2	2	2	2	2	2	2	2	2	2
27	V		11	3	6	10	10	10	10	10	10	10	10	10	10	R							
28	IV		8	0	0	0	0	0	0	0	0	0	0	0	0	0	0	0	0	0	0	0	0
29	IV		7	0	0	0	0	0	0	0	0	0	0	0	0	0	0	0	0	0	0	0	0
30	V		10	1	1	1	1	1	1	1	1	1	1	1	1	1	1	1	1	1	1	1	1
31	VI		12	9	9	9	9	9	9	9	9	9	9	9	9	9	9	R					
32	VI		12	2	8	12	R																
33	VI		12	2	3	3	3	3	3	3	3	3	3	3	3	3	3	3	3	3	3	3	
34	V		9	5	5	5	5	5	5	5	6	7	8	R									
35	V		10	0	1	1	1	3	3	1	1	1	1	1	1	1	1	1	1	1	1	1	
36	V		11	0	1	1	1	1	1	1	1	1	1	1	1	1	1	1	1	1	1	1	
37	VI		12	8	10	10	10	10	10	11	R												
38	V		10	0	0	0	0	0	0	0	0	0	0	0	0	0	0	0	0	0	0	0	
39	V		9	0	0	0	0	2	2	2	2	2	2	2	2	2	2	2	2	2	2		
40	V		11	6	8	9	9	9	10	10	R												
41	V		9	0	0	0	0	0	0	0	0	0	0	0	0	0	0	0	0	0	0		
42	VI		12	2	2	2	2	2	2	2	2	2	2	2	2	2	2	2	2	2	2		
43	IV		7	4	4	4	4	2	3	3	3	3	3	3	3	3	3	3	3	3	3		
44	IV		6	0	0	0	0	0	0	0	0	0	0	0	0	0	0	0	0	0	0		
45	VI		12	6	6	6	7	7	8	R													
46	VI		12	7	12	12	12	12	R														
47	V		11	3	4	4	5	5	5	5	5	5	5	5	5	5	5	5	R				
48	V		11	0	1	2	2	2	2	2	2	2	2	2	2	2	2	2	2	2	2		

## Data Availability

The patients' data used to support the findings of this study are included within the paper.
